# Sleep and cardiac autonomic modulation in older adults: Insights from an at‐home study with auditory deep sleep stimulation

**DOI:** 10.1111/jsr.14328

**Published:** 2024-09-02

**Authors:** Stephanie Huwiler, M. Laura Ferster, Luzius Brogli, Reto Huber, Walter Karlen, Caroline Lustenberger

**Affiliations:** ^1^ Neural Control of Movement Lab, Department of Health Sciences and Technology Institute of Human Movement Sciences and Sport, ETH Zurich Zurich Switzerland; ^2^ Mobile Health Systems Lab, Department of Health Sciences and Technology Institute of Robotics and Intelligent Systems, ETH Zurich Zurich Switzerland; ^3^ Neuroscience Center Zurich (ZNZ) University of Zurich and ETH Zurich Zurich Switzerland; ^4^ Center of Competence Sleep & Health Zurich University of Zurich Zurich Switzerland; ^5^ Child Development Centre, University Children's Hospital University of Zurich Zurich Switzerland; ^6^ Department of Child and Adolescent Psychiatry and Psychotherapy, Psychiatric Hospital Zurich University of Zurich Zurich Switzerland; ^7^ Institute of Biomedical Engineering Universität Ulm Ulm Germany

**Keywords:** aging, auditory stimulation, cardiac autonomic regulation, decentralized study, slow waves

## Abstract

The autonomic nervous system regulates cardiovascular activity during sleep, likely impacting cardiovascular health. Aging, a primary cardiovascular risk factor, is associated with cardiac autonomic disbalance and diminished sleep slow waves. Therefore, slow waves may be linked to aging, autonomic activity and cardiovascular health. However, it is unclear how sleep and slow waves are linked to cardiac autonomic profiles across multiple nights in older adults. We conducted a randomized, crossover trial involving healthy adults aged 62–78 years. Across 2 weeks, we applied auditory stimulation to enhance slow waves and compared it with a SHAM period. We measured sleep parameters using polysomnography and derived heart rate, heart rate variability approximating parasympathetic activity, and blood pulse wave approximating sympathetic activity from a wearable. Here, we report the results of 14 out of 33 enrolled participants, and show that heart rate, heart rate variability and blood pulse wave within sleep stages differ between the first and second half of sleep. Furthermore, baseline slow‐wave activity was related to cardiac autonomic activity profiles during sleep. Moreover, we found auditory stimulation to reduce heart rate variability, while heart rate and blood pulse wave remained unchanged. Lastly, within subjects, higher heart rate coincided with increased slow‐wave activity, indicating enhanced autonomic activation when slow waves are pronounced. Our study shows the potential of cardiac autonomic markers to offer insights into participants' baseline slow‐wave activity when recorded over multiple nights. Furthermore, we highlight that averaging cardiac autonomic parameters across a night may potentially mask dynamic effects of auditory stimulation, potentially playing a role in maintaining a healthy cardiovascular system.

## 1 INTRODUCTION

Beyond sleep's function in promoting a healthy brain, sleep is also recognized as a state for the body to recover. Insufficient sleep quantity and quality have been associated with short‐ and long‐term health consequences (Medic et al., 2017), and heightened cardiovascular disease risk factors (Grandner et al., 2016).

Advancing age is not only the primary risk factor for the onset of cardiovascular diseases (North & Sinclair, 2012), but healthy aging is accompanied by increased sleep fragmentation, reduced polysomnography‐derived sleep duration (Espiritu, 2008), and profoundly diminished deep non‐rapid eye movement (NREM) sleep (Landolt et al., 1996). This decline in deep NREM sleep may represent a pivotal link between deteriorating cardiovascular health and the aging process.

Cardiovascular activity is mainly regulated through the autonomic nervous system (Cabiddu et al., 2012; Mancia, 1993), exhibiting parasympathetic predominance (Brandenberger et al., 2001; Jurysta et al., 2003) and sympathetic withdrawal (Somers et al., 1993) during NREM sleep, and opposite profiles during rapid eye movement (REM) sleep (Somers et al., 1993). Beyond the influence of aging on sleep physiology, the aging process has been associated with decreased parasympathetic activity during sleep, whereas the dynamics of the cardiac autonomic regulation remain likely unchanged (Jurysta et al., 2005). However, most of the research has been conducted in single‐night studies, and it is unclear how cardiac autonomic modulation characteristics change between different nights.

Recently, slow waves, the dominant brain oscillations of deep NREM sleep, have been functionally linked to enhanced parasympathetic activity (Diep et al., 2022; Grimaldi et al., 2019), but whether the age‐related decline in slow waves in healthy older adults (Landolt et al., 1996) is epiphenomenal or functionally involved in the proposed diminished parasympathetic modulation remains to be explored. Over the last few years, auditory stimulation has gained attention to selectively modulate slow waves during sleep (Diep et al., 2020; Grimaldi et al., 2019; Ngo et al., 2013), with potential applications in remote and long‐term settings (Lustenberger et al., 2022). However, most studies have investigated only single nights of auditory deep sleep stimulation within the sleep lab environment in healthy young men.

Here, we analysed the cardiovascular secondary outcomes of a randomized, crossover clinical trial applying auditory sleep stimulation administered in two approaches (continuous and windowed) over several weeks in the homes of healthy older participants aged between 60 and 85 years. We found that auditory stimulation can enhance slow‐wave activity (SWA) in this population over the 2 weeks of intervention. The more detailed primary outcomes of this clinical trial were reported elsewhere (Lustenberger et al., 2022). We first characterized cardiac autonomic modulation indirectly assessed through heart rate (HR), HR variability (HRV) as a marker for parasympathetic activity and blood pulse wave (BPW) estimating sympathetic activity across sleep stages in multiple nights per participant. Furthermore, we investigated whether individual fluctuations in SWA levels across multiple nights are correlated with the cardiac autonomic modulation profiles. In our previous publication (Lustenberger et al., 2022), we were able to distinguish strong and weak responders to auditory stimulation based on each individual baseline SWA, which likely reflects trait‐like characteristics. Thus, we wanted to further elucidate whether cardiac autonomic modulation between these two groups also differs across sleep stages. Next, we investigated whether multiple nights of auditory stimulation and stimulation characteristics (such as the number of stimulations within a sleep epoch) influence cardiac autonomic modulation. Lastly, we explored whether baseline SWA, as indicated by an index of responsiveness to auditory stimulation (respond index; Lustenberger et al., 2022), interacts with cardiac autonomic activity.

## 2 METHODS

### 2.1 Participants

We enrolled 33 participants in this double‐blinded, randomized–controlled trial (registered at ClinicalTrials.gov: NCT03420677). Here, we analysed the data of a subgroup all having completed the study protocol and who received auditory up‐phase slow‐wave stimulation over multiple weeks (see Figure S1 for an overview of participant enrolment). Participants were recruited from the local community using advertisements and information at meet‐ups. The here‐reported analysis serves as a secondary analysis to the primary outcomes that have been previously published (Lustenberger et al., 2022). Participants were non‐smokers, of good general health, and lived in a stable home situation. Participants taking on‐label sleep medication, and suffering from a diagnosed neurological, internal, psychological or sleep disorder were excluded as they did not meet the inclusion criteria. Additionally, participants who did not pass a simple audiometry with the headphones used for the stimulation (50 dB on both ears) were further excluded. The study was approved by the Swiss Agency for Therapeutic Products Swissmedics and the Cantonal Ethics Committee Zurich. All participants provided written informed consent before participation and received monetary compensation for their participation. The study was conducted in accordance with the Declaration of Helsinki.

### 2.2 Experimental procedure

**FIGURE 1:**
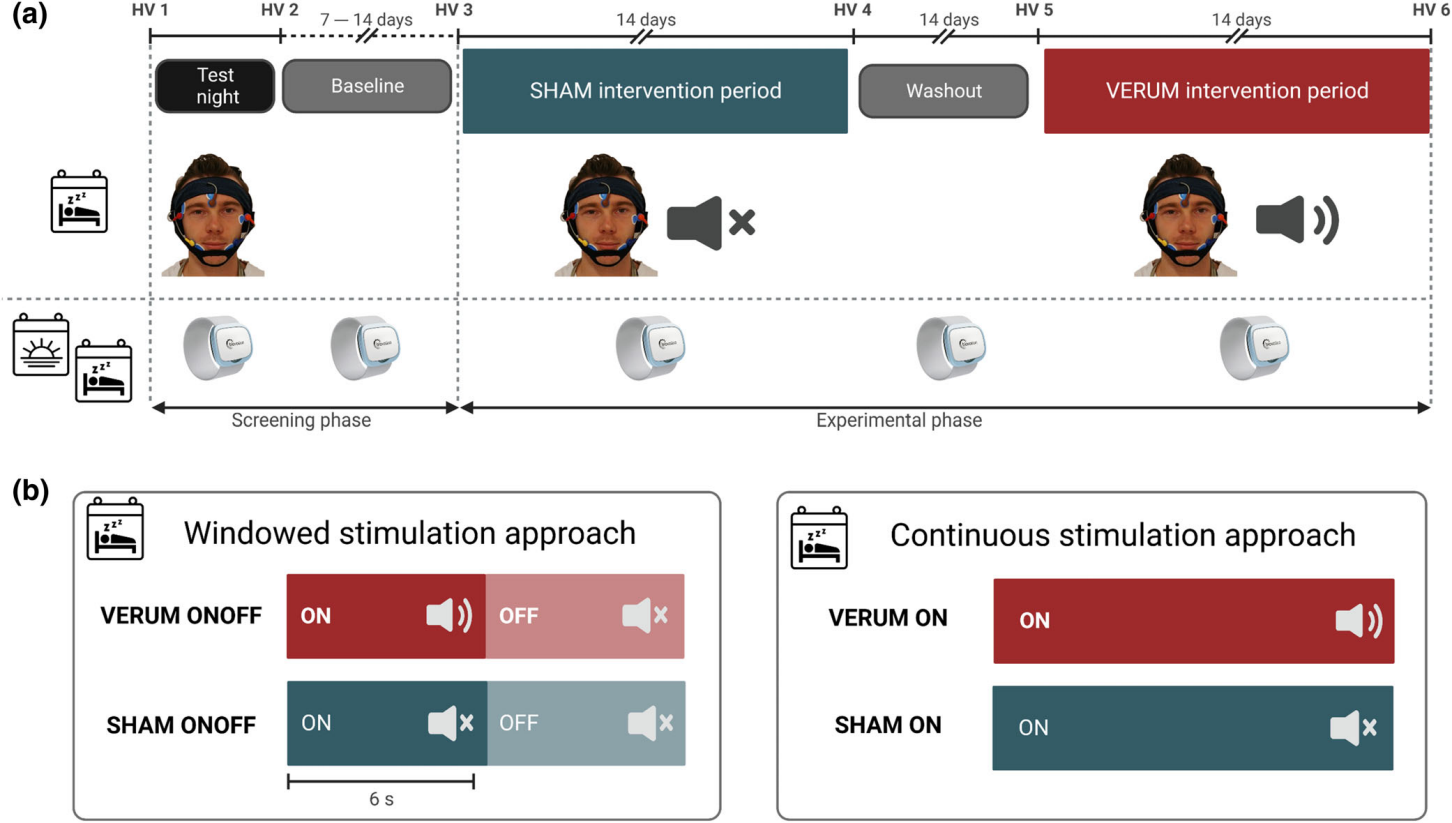
Experimental procedure of study. (a) Participants underwent an initial screening phase consisting of a test night to assess eligibility and to familiarize participants to the intervention device (MHSL‐Sleepband, MHSL‐SB), to the wearable Everion, and to additional questionnaires. After a baseline period, the experimental phase consisted of two, 2‐week intervention periods interleaved with a 2‐week washout. Either a SHAM condition (no auditory stimulation) or VERUM (auditory stimulation) were conducted in a randomized, crossover design. During the complete periods, participants were wearing the wearable to record physiological signals. During the night, participants additionally wore the MHSL‐sleep band to record electroencephalography (EEG; Fpz‐A2), left and right electrooculography (EOG), and chin electromyography (EMG). (b) Auditory stimulation protocol. Stimulation was either delivered in a windowed 6‐s ON followed by 6‐s OFF design (windowed stimulation approach), or continuously throughout stable non‐rapid eye movement (NREM) sleep (whenever stimulation conditions were met and the up‐phase of a detected slow wave was reached/continuous stimulation approach). For each intervention period, both stimulation types were delivered: 7 days windowed and 7 days continuous stimulation (note that during SHAM stimulation, tones were muted) in a randomized design (see Lustenberger et al., 2022 for details).

An overview of the experimental procedure is summarized in Figure 1(a). All participants underwent an initial screening phase consisting of a phone screening to verify eligibility and a first home visit. During this first home visit, participants answered several demographic questionnaires, and were instructed on how to use the intervention device (MHSL‐SB) and the wearable (Everion, Biovotion AG, Zurich, Switzerland), as well as how to answer the daily questionnaires on the phone. Furthermore, participants performed a basic audiometry. After this home visit, participants slept wearing the MHSL‐SB and the wearable to get used to the interventional protocol. The following day, if participants fulfilled all inclusion criteria, they were given the option to decide about participating in the experimental period. Thereafter, a baseline period followed to get the participants habituated to the mobile phone questionnaires, assessments and the wearable. After this baseline period, the experimental phase, consisting of two, 2‐week intervention periods separated by a 2‐week washout period, started. During the experimental period, participants wore the wearable continuously and, during the night, participants were additionally wearing the MHSL‐SB to record electroencephalography (EEG), electrooculography (EOG) and electromyography (EMG) data, and received the allocated intervention (either no stimulation: SHAM; or auditory stimulation: VERUM). During the intervention periods, both stimulation conditions were further subdivided into a windowed and a continuous stimulation approach, as described in more detail (Lustenberger et al., 2022) and illustrated in Figure 1(b). The stimulation conditions were assigned based on a randomized‐counterbalanced design, and gender was considered additionally while randomization was performed. Furthermore, participants answered a sleep diary and mood assessments daily (Lustenberger et al., 2022). During the beginning and end of each intervention period, another home visit took place where participants completed a cognitive task battery and answered quality‐of‐life questionnaires. In the middle of the intervention period, members of the study team exchanged the intervention device.

### 2.3 Auditory stimulation protocol

A single‐channel EEG (Fpz‐A2), two EOG and two EMG channels were recorded using single‐use auto‐adhesive electrodes (Neuroline 720, Ambu A/S, DK), acquired through the MHSL‐SB, a self‐developed system for real‐time sleep recording and processing with an implemented option to deliver auditory stimulation (Ferster et al., 2019). These sleep biosignals were recorded at 250 Hz. The Fpz‐A2 signal was used to trigger the real‐time auditory stimulation logic, which consisted of notch‐filtering at 50 Hz and NREM sleep detection based on power ratios in different frequency bands (low delta: 2–4 Hz; high delta: 3–5 Hz; and high beta: 20–30 Hz). Furthermore, during the first 10 min of detected and uninterrupted NREM sleep, an individual beta threshold was automatically calculated. After the 10‐min NREM baseline period, stimulation was enabled. Stimulation occurred if delta power exceeded a predefined threshold, beta power was below the individual beta threshold, and stable NREM sleep was detected (at least 3 min NREM sleep before stimulation). Additionally, a first‐order phase locked loop was implemented in the MHSL‐SB to allow targeting the up‐phase (here: 45°) of ongoing slow waves. The minimal inter‐tone interval was set to 500 ms. Auditory stimuli consisted of 50‐ms bursts of pink noise with a variable volume of stimulation ranging from a minimal volume of 46 dB, a default volume of 52 dB, and increased up to 60 dB while no arousal occurred. Stimulation was interrupted if signs of arousals were detected (Lustenberger et al., 2022).

### 2.4 Cardiovascular recording and synchronization with EEG

The wearable recorded among others accelerometer data, HR, HRV and BPW. All data were sampled at 51.2 Hz except the accelerometer data at 50 Hz, before automatic down‐sampling to 1 Hz. HR was calculated based on a 10‐s moving average of the detected heartbeats measured through photoplethysmography. HRV was calculated as the root‐mean‐square of successive differences (RMSSD) of the inter‐beat intervals within a 5‐min sliding window. BPW was calculated as a 5‐min moving average based on a proprietary algorithm by Biovotion AG, using the shape, rhythmicity and speed of the photoplethysmographic‐derived blood pressure wave for its calculations. High rhythmicity is related to increased BPW. Each datapoint included a quality index between 0 and 100, with a quality above 50 indicating compliance with medical‐grade data quality. Therefore, only data that coincide with medical grade quality were used for this analysis.

To synchronize the biosignal recording of the MHSL‐SB with the wearable data, we implemented a semi‐automatic synchronization algorithm within MATLAB (R2021a, The MathWorks, Natick, MA, USA) aligning the activity data of the wearable, which is a classically used tool for defining wake and sleep periods based on accelerometer data (Dick et al., 2010), with the EEG spectral power of 26.25–32.00 Hz, which has been proposed to detect muscular artefacts within the EEG (Brunner et al., 1996) recorded through the MHSL‐SB, termed here as movement beta power. First, the continuous 2‐week measurement period of the wearable accelerometer recording was split into single nights ranging from 20:00 hours to 10:00 hours the next morning. Afterward, the time spent in bed was roughly calculated based on periods of activity or inactivity that lasted longer than 5 min each. Based on previous and following periods of activity or inactivity, periods were labelled as wake, rest, sleep, or arousal. Finally, information from the sleep diary that was completed the next morning by the participants was considered for the classification algorithm. Bedtime was defined as the first wearable timestamp within the first period identified as sleep, and sleep offset as the last wearable timestamp of the last labelled sleep period.

**FIGURE 2:**
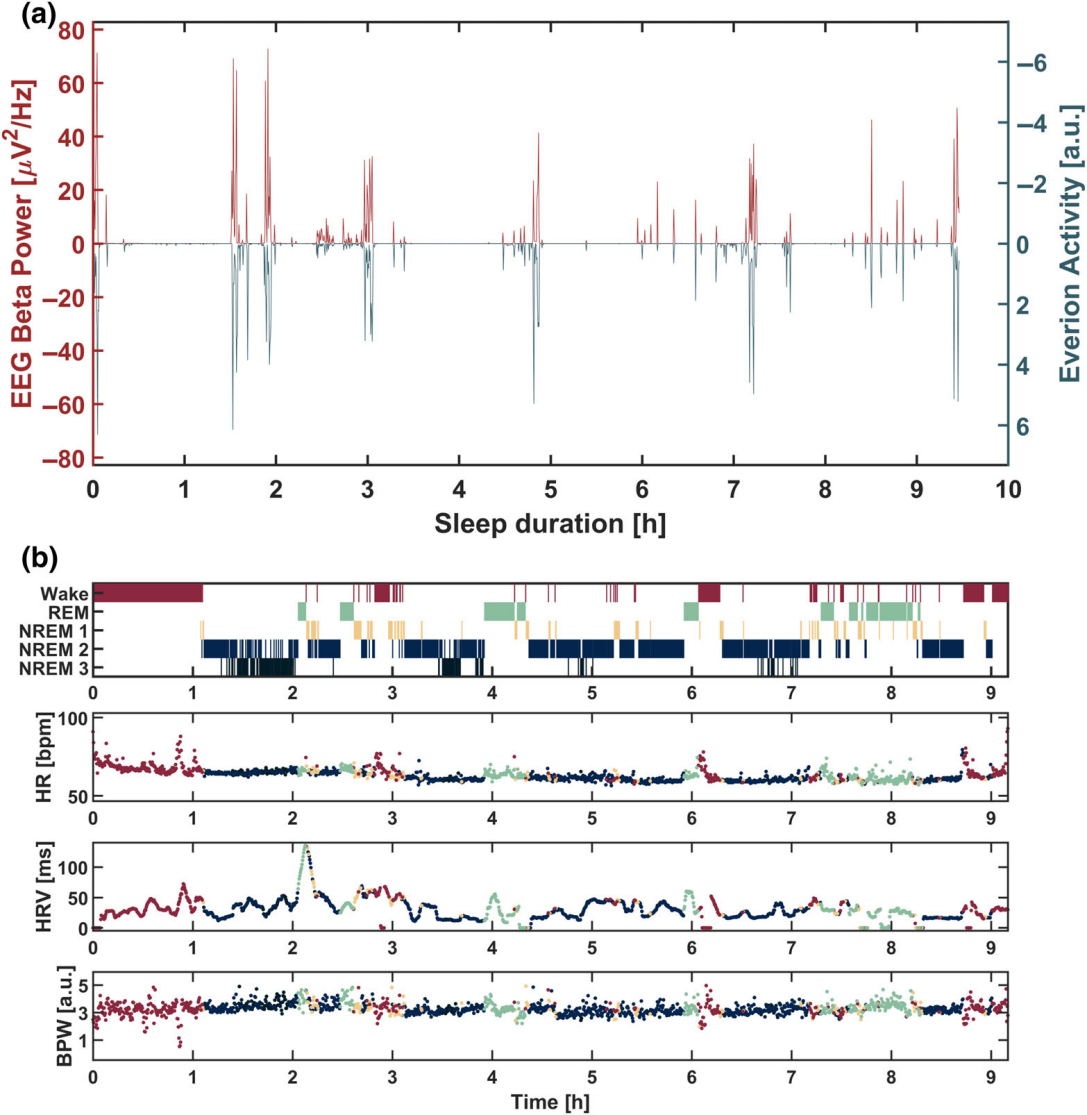
Synchronization of wearable with sleep electroencephalography (EEG). (a) EEG power in the beta frequency band (26.25–32.00 Hz, termed movement beta) in red (positive scale on the left), and accelerometer (activity) data of Everion wearable in blue‐green (inverted scale, negative values on the right). The plot shows a well‐synchronized night between sleep EEG and wearable accelerometer data. (b) Exemplary night of the sleep hypnogram on top (dark red: wake; orange: non‐rapid eye movement [NREM] sleep stage 1 [NREM1]; blue: NREM2; dark‐blue: NREM3; green: rapid eye movement [REM] sleep). The second subplot shows heart rate (HR), the third plot shows HR variability (HRV), here as the root‐mean‐square of successive differences (RMSSD) index, and the fourth subplot shows blood pulse wave (BPW).

To align the EEG with the wearable signal, the wearable accelerometer data were averaged into 20‐s segments to match the length of sleep staging epochs. Then, the calculated sleep offset time was synchronized with the last point within the EEG recording with movement beta power below the 92nd percentile. Thereafter, for both the wearable and the EEG data, each datapoint was assigned either a 0 if its value was below the 92nd percentile of the complete recording, or 1 if it was above the 92nd percentile. These two signals were cross‐correlated within MATLAB using the function *finddelay* of the Signal Processing Toolbox with a maximal lag of 60 min. Each night was then visually inspected, and the start points were corrected, if necessary, until both signals were aligned. A successfully synchronized night is illustrated in Figure 2(a). If synchronization figures did not meet expectations (e.g. too high variance between the EEG movement beta power and wearable accelerometer signal), nights were marked and excluded for further analyses. Generally, one participant had to be excluded because of the too high variance and, in addition, another 98 nights were excluded either because of bad quality EEG data (see next paragraph), missing wearable data, or insufficient synchronization quality. All recorded wearable signals were then averaged again into 20‐s periods matching the EEG signals recorded with the MHSL‐SB that were averaged into 20‐s epochs. Only wearable epochs with data quality higher than 50 were considered for further analyses. Missing datapoints and outliers detected through a 5‐min moving median were interpolated using the piecewise cubic hermite interpolating polynomial method.

### 2.5 Hybrid sleep scoring and EEG analysis

Sleep was automatically scored using a deep learning classification algorithm followed by a manual expert review of classifications with low confidence scores within 20‐s epochs. Details of the algorithm are described in Lustenberger et al. (2022). All nights with more than 20 min of bad EEG signal quality, only partial recordings present, with electrodes not yet connected when recording started or disconnected before recording, or recording stopped for more than 20 min were excluded from all analyses.

First, the EEG data were high‐pass filtered at 0.5 Hz and low‐pass filtered at 40 Hz using FIR filters provided by the EEGLAB toolbox for MATLAB (The MathWorks, Natick, MA, USA). Thereafter, the EEG was either analysed with a consecutive analysis or ON–OFF analysis method. For both methods, the spectral power of 6‐s epochs was calculated using the MATLAB *pwelch* function with a 4‐s window length with 50% overlap. We analysed lowSWA, that is EEG power in the low slow‐wave frequency range (0.75–1.25 Hz), as our primary outcome variable to investigate the main effect of auditory stimulation on brain oscillations. This is among the most consistently reported findings in previous in‐lab studies using auditory slow‐wave sleep modulation. A detailed description of all EEG processing steps has been published in Lustenberger et al. (2022).

For the analyses within NREM sleep (here defined as NREM2 + 3 sleep), only epochs that were considered as NREM by both the MHSL‐SB device algorithm and the hybrid sleep scoring were included. Within these epochs, spectral power was calculated using the function *pwelch* with 4‐s window length with 50% overlap. Epochs being identified as outliers based on the *isoutlier* function of MATLAB were also excluded as well as epochs being identified as outliers by a semi‐automatic artefact rejection method (Lustenberger et al., 2015).

Arousals were automatically detected during hybrid scored NREM1–3 sleep and REM sleep using a published, open‐source algorithm (Fernández‐Varela et al., 2017). In short, the algorithm uses one raw EEG and EMG derivation, and detects arousal‐based EEG power in the alpha (8–12 Hz) and beta (> 16 Hz) bands. Epochs containing arousals were not included in the analysis.

### 2.6 Statistics and reproducibility

Statistical analyses were conducted in RStudio (R Foundation for Statistical Computing, Vienna, Austria) version 1.2.5033. To analyse the effects of condition and/or night, we employed robust linear mixed‐effect models using the R‐package *robustlmm*. Participants could be distinguished into strong responders and weak responders (respond index) to auditory sleep stimulation (Lustenberger et al., 2022). Thus, to investigate whether respond index influences the cardiac autonomic parameters, we included respond index in the models as well (see Lustenberger et al., 2022 for details about responder analysis). To derive *F*, *p*‐values, nominator and denominator degrees of freedom, we used the Kenward–Roger approximation. To calculate the differences in the cardiac autonomic parameters between sleep stages, we employed linear mixed‐effect models using the R‐package *lme4*. Post‐hoc *p*‐values were derived using the Satterthwaite method implemented within *lmerTest*. Comparisons between sleep stages and correction for multiple comparisons were performed using the R package *emmeans* and the Hochberg method. All models included the nested random factor of subject and condition, fixed factors included (depending on the analysis) condition, night, sleep stage, respond index. The factor night represents the variability between nights. Moreover, to account for cardiovascular changes within single nights, we split each night into two halves and call this factor *nightpart*.

To further investigate whether night‐to‐night variance within subjects influences cardiac autonomic parameters, we performed repeated measures correlation using the package *rmcorr*, which calculates the intra‐individual association between two measures considering repeated measures. To calculate the difference of the stimulation to the SHAM stimulation period, only epochs within scored NREM2 + 3 sleep and where the MHSL‐SB device recognized NREM sleep were taken to calculate the mean value for each night first, and afterwards to calculate the average within the SHAM ON and SHAM ONOFF periods. The average of each night of the VERUM ON and VERUM ONOFF condition within NREM2 + 3 and detected NREM sleep by the MHSL‐SB were then used to calculate the relative difference to the average SHAM value of the matching condition (e.g. VERUM ON was compared with SHAM ON). We considered *p*‐values < 0.05 as significant. Figures were plotted within either MATLAB or R using *ggplot* and *ggprism*.

## 3 RESULTS

We here report insights on derived cardiac autonomic activity data collected as secondary outcomes of a randomized, crossover clinical trial investigating the effects of auditory deep sleep stimulation over multiple nights. All participants completed twice, 2 weeks of at‐home EEG monitoring while auditory or SHAM stimulation was applied. Out of the 16 healthy older adults receiving the allocated intervention, one participant had to be excluded as the wearable data synchronization with the EEG did not work sufficiently, and another participant as there was EEG data loss during a complete VERUM on period. The analysed subgroup included 14 participants (six females, age: 70.09 ± 4.98 years; Figure S1) and a total of 269 nights (see Table S1 as an overview of the number of included nights per condition).

### 3.1 Cardiac autonomic parameters differ between sleep stages

To investigate the influence of sleep stage on cardiac autonomic parameters, we calculated the mean value of HR, HRV and BPW within each sleep stage (wake, NREM2, NREM3, REM) for each sleep period. Because NREM1 can be viewed as a transition sleep stage, we did not include NREM1 in our analyses (Colten & Altevogt, 2006).

**FIGURE 3:**
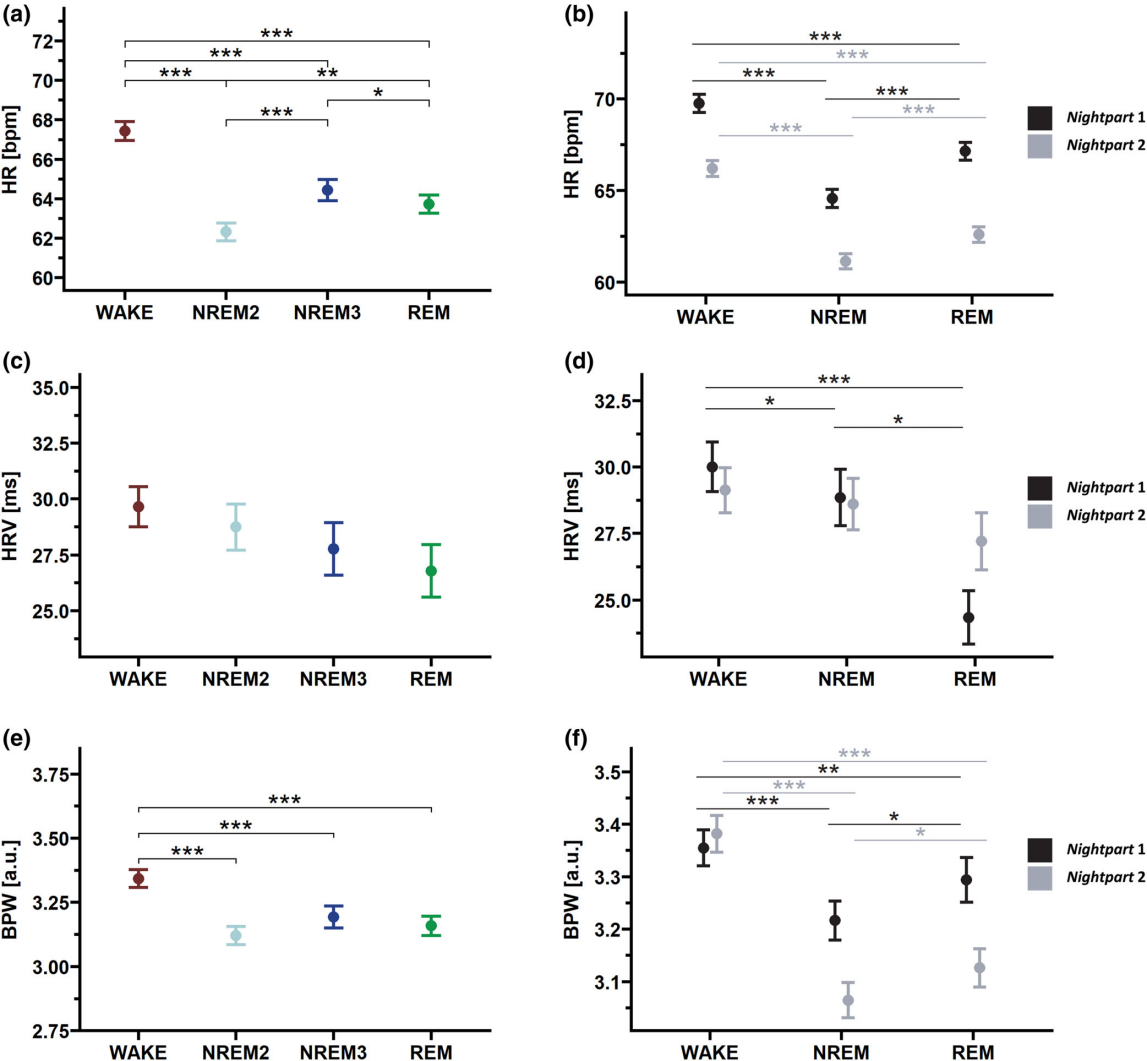
Sleep stage differences for heart rate (HR), HR variability (HRV) and blood pulse wave (BPW). (a,c,e) Post‐hoc pairwise comparisons between sleep stage for the variables HR, HRV (calculated as root‐mean square of successive differences [RMSSD]) and BPW across the complete night were derived from linear mixed‐effect models entering sleep stage and night as fixed factors, and the nested random factors subject and condition. Included sleep stages are wake, non‐rapid eye movement (NREM) sleep stage 2 (NREM2), NREM Stage 3 (NREM3) and rapid‐eye movement (REM) sleep. (b,d,f) HR, HRV and BPW for *nightpart* 1 and *nightpart* 2. Post‐hoc pairwise comparisons between sleep stage for the variables HR, HRV (calculated as RMSSD) and BPW across *nightpart* 1 (first half, in black) and *nightpart* 2 (second half, in grey) were derived from linear mixed‐effect models entering sleep stage and night as fixed factors, and the nested random factors subject and condition. Included sleep stages are wake, NREM sleep stages 2 and 3 together (NREM), and REM sleep. All data are presented as mean ± standard error of the mean. *p < 0.05; **p < 0.01; ***p < 0.001.

There was a significant influence of sleep stage (*F*_3,1070_ = 113.42, *p* < 0.001) on HR while adding night as a control factor (*F*_13,1070_ = 5.01 p < 0.001). Post‐hoc comparison of HR between sleep stages is shown in Figure 3(a), demonstrating the lowest HR occurring during NREM2. We found no significant influence of sleep stage on HRV (*F*_3,1070_ = 1.82, *p* = 0.143), as shown in Figure 3(c); however, night significantly affected HRV (*F*_13,1070_ = 2.33, *p* = 0.005). Analysing BPW as a marker for sympathetic activity, we found a significant effect of night (*F*_13,1071_ = 2.20, *p* = 0.008) and sleep stage (*F*_3,1070_ = 40.54, *p* < 0.001) with decreased BPW for all sleep stages compared with wake, as illustrated in Figure 3(e).

Because the length of NREM3 sleep decreases throughout the aging process (Mander et al., 2013), and NREM2 and NREM3 sleep can be considered as a continuum, we also investigated how cardiac autonomic parameters vary between NREM sleep (NREM2 + 3 combined), REM sleep, and wake. These results are shown in Figure S2.

### 3.2 Cardiac autonomic modulation differs between *nightparts*

Evidence shows that HR (Viola et al., 2002) and HRV (Brandenberger et al., 2001; Jurysta et al., 2003) are influenced by sleep, as HR tends to be higher during the beginning of the night compared with the end. Because there is a higher proportion of REM sleep and less deep NREM sleep towards the end of the night, taking the mean HR for each sleep stage across the complete night is likely not a fair comparison. Thus, we investigated whether *nightpart* (splitting the night into two halves) changes the sleep stage influence on HR, HRV and BPW. Again, we considered NREM2 and NREM3 as one sleep stage NREM because during the end of the night, the proportion of NREM3 is decreasing and there can be no single NREM3 epoch within *nightpart* 2. We found no significant interaction between *nightpart* and sleep stage on HR (*F*_2,1483_ = 0.44, *p* = 0.647), but *nightpart* had a significant effect on HR (*F*_1,1486_ = 427.11, *p* < 0.001), with lower HR in *nightpart* 2. Furthermore, we observed a significant interaction between sleep stage and *nightpart* on HRV (*F*_2,1483_ = 5.34, *p* = 0.005), indicating that HRV only differed between sleep stages in *nightpart* 1. Lastly, we observed a significant interaction between sleep stage and *nightpart* on BPW (*F*_2,1483_ = 17.72, *p* < 0.001), indicating a more pronounced decrease of BPW within NREM and REM during *nightpart* 2.

Next, we analysed how cardiac autonomic parameters differ between sleep stages within each *nightpart* separately. Within *nightpart* 1, we found sleep stage differences in HR (*F*_2,767_ = 107.06, *p* < 0.001), with the lowest HR during NREM sleep. HRV was significantly different between sleep stages (*F*_2,757_ = 10.36, *p* < 0.001), and was highest during wake and lowest during REM sleep. Finally, sleep stages also significantly affected BPW (*F*_2,769_ = 18.52, *p* < 0.001), with the lowest BPW during NREM sleep. Within *nightpart* 2, sleep stage significantly influenced HR (*F*_2,790_ = 171.65, *p* < 0.001), with lowest HR during NREM sleep, and BPW (*F*_(2,791)_ = 107.41, *p* < 0.001), again with lowest values during NREM sleep. However, there was no difference in HRV between sleep stages within *nightpart* 2 (*F*_2,785_ = 2.77, *p* = 0.064). All significant pairwise comparisons between sleep stages for *nightpart* 1 and *nightpart* 2 are shown in Figure 3(b,d,f).

### 3.3 SWA is positively correlated to HR but to no other cardiac autonomic parameter

**FIGURE 4:**
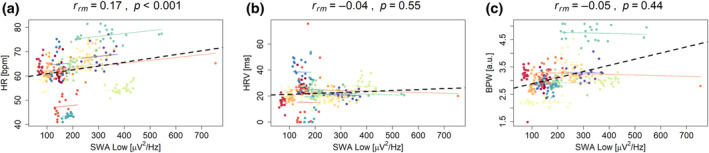
Repeated measures correlations. (a) Between the mean low slow‐wave activity (SWA) within non‐rapid eye movement (NREM) sleep (scored and detected by the MHSL‐SB device) and the average heart rate (HR) within the same epochs, for each night. (b) Between the mean HR variability (HRV) within NREM sleep (scored and detected by the MHSL‐SB device) within the same epochs, for each night. (c) Between the mean blood pulse wave (BPW) within NREM sleep (scored and detected by the MHSL‐SB device) within the same epochs, for each night. The *r*_rm_ values refer to the within‐subject correlations that are also represented through the dotted coloured lines. The dotted black line represents the overall regression line.

Because of the inter‐night variability in lowSWA within single subjects, as has been previously published (Lustenberger et al., 2022), we wanted to investigate the relationship between lowSWA and cardiac autonomic variables. We thus employed repeated measures correlations between mean lowSWA and mean HR, mean HRV and mean BPW within NREM sleep for each night. We found significant positive repeated measures correlations between lowSWA and HR (Figure 4a), indicating that within subjects, nights with higher lowSWA were associated with higher HR. The repeated measures correlations analyses revealed no significant correlation between HRV and lowSWA (*r*_rm_ = −0.04, *p* = 0.55), and BPW and lowSWA (*r*_rm_ = −0.05, *p* = 0.44; Figure 4b,c).

### 3.4 Baseline lowSWA level influences cardiac autonomic parameters during sleep

**FIGURE 5:**
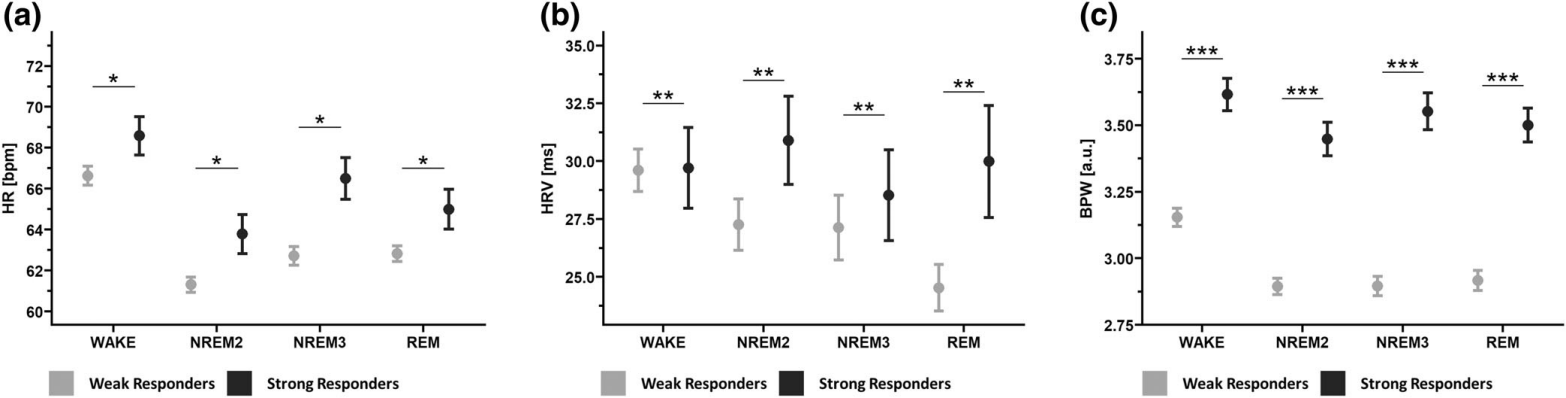
Interaction of respond index and sleep stage for heart rate (HR), HR variability (HRV) and blood pulse wave (BPW): within each sleep stage, separated for strong responders and weak responders. The *p*‐values characterize the interaction between sleep stage and respond index of the linear mixed‐effect models. All data are presented as mean ± standard error of the mean. *p < 0.05; **p < 0.01; ***p < 0.001.

There are not only within‐subject differences in lowSWA between nights but also between subjects. In our participant cohort, participants could be distinguished into strong responders and weak responders (respond index) to auditory stimulation based on their baseline lowSWA level (Lustenberger et al., 2022). We now wanted to explore whether cardiac autonomic parameters also differ based on the trait‐like baseline lowSWA level. Out of the included participants, six could be classified as strong responders (four females; age: 65.56 ± 2.29 years) and eight as weak responders (two females; age: 73.48 ± 3.42 years). We found a significant interaction between respond index and sleep stage (NREM2, NREM3, REM and wake) on HR (*F*_3,1066.9_ = 2.82, *p* = 0.038). Furthermore, we found a significant interaction between respond index and sleep stage on HRV (*F*_3,1067_ = 4.45, *p* = 0.004), and a significant interaction between respond index and sleep stage on BPW (*F*_3,1067_ = 5.69, *p* < 0.001). All mean values of HR, HRV and BPW for each sleep stage for both groups, the strong responders and weak responders, are illustrated in Figures 5 and S2(b,d,f). Note that the *p*‐values refer to the interaction between sleep stage and respond index.

### 3.5 Auditory stimulation affects HRV while leaving HR and BPW unchanged

Next, we wanted to elucidate whether slow‐wave enhancement through auditory stimulation also influences cardiac autonomic parameters. Therefore, we first investigated whether auditory stimulation influences HR, HRV and BPW within NREM sleep. We employed two different stimulation approaches (see Section 2 and Lustenberger et al., 2022, for detailed description): a continuous approach, wherein stimulation occurred whenever specific conditions were met and a slow‐wave up‐phase was detected; and a windowed approach, involving alternating periods of 6‐s ON followed by 6‐s OFF, during which stimulation was active solely within the designated ON window. Because the two different stimulation approaches increased lowSWA differently (Lustenberger et al., 2022), we here separated the continuous and the windowed approach. We found no significant effect of stimulation condition on HR (*F*_1,10_ = 0.76, *p* = 0.403), HRV (*F*_1,10_ = 2.03, *p* = 0.186) and BPW (*F*_1,10_ = 0.10, *p* = 0.762) for the windowed stimulation approach. For the continuous stimulation approach, we observed a significant decrease in HRV during auditory stimulation compared with SHAM (*F*_1,10_ = 5.21, *p* = 0.045). Additionally, we found no effect of stimulation condition on HR (*F*_1,11_ = 0.59, *p* = 0.459) and BPW (*F*_1,10_ = 1.53, *p* = 0.244).

**FIGURE 6:**
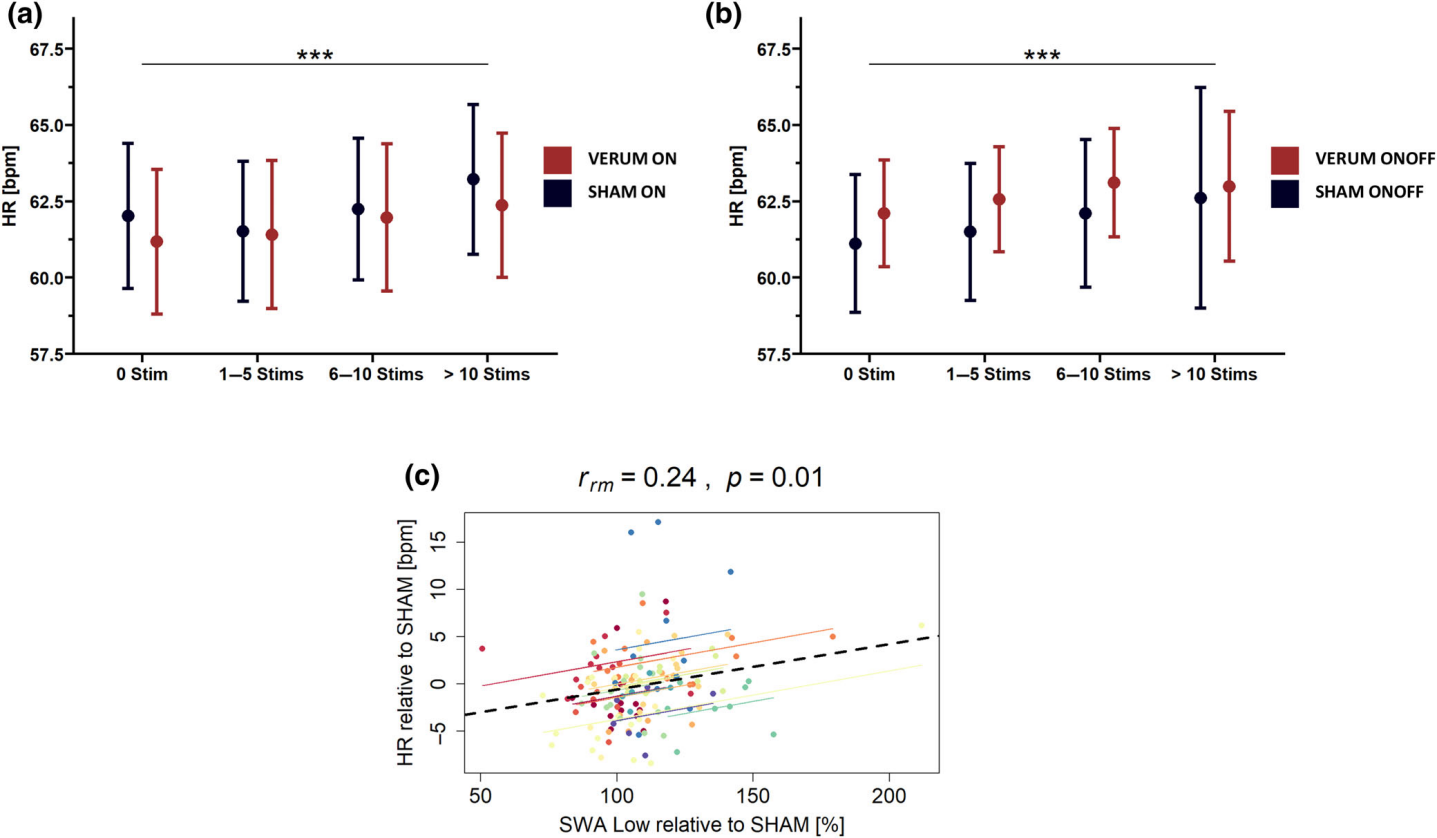
Effects of auditory deep sleep stimulation on heart rate (HR) for the continuous stimulation (ON) and windowed (ONOFF) approach. (a,b) Effects of number of stimulations (separated in bins of 0, 1–5, 5–10, > 10 stimulations) and condition on HR. Note that *p*‐values refer to factor stimulation bin. (c) Repeated measures correlations between the increase in low slow‐wave activity (SWA) for each VERUM night compared with the average SHAM period (see Methods for details) compared with the relative change in HR between VERUM and SHAM. The *r*_rm_ value refers to the within‐subject correlation that is also represented through the dotted coloured lines. The dotted black line represents the overall regression line. ***p < 0.001.

Next, we analysed whether the number of stimulations influences HR within an epoch. As HR is the only variable measured with the wearable with sufficient time resolution for this analysis, we do not report HRV or BPW measures here. We analysed all NREM epochs of 20 s and grouped all stimulations into bins of 0 stims, 1–5 stims, 6–10 stims or more than 10 stims, and calculated the mean HR per night per subject within each of the stimulation bins for the continuous and windowed approach separately (Figure 6a,b). We found no significant effect of condition (*F*_1,12_ = 0.63, *p* = 0.442) on HR within the continuous approach, and neither for the windowed approach (*F*_1,11_ = 0.26, *p* = 0.621). However, stimulation bin significantly influenced HR for the continuous (*F*_3,469_ = 9.20, *p* < 0.001) and the windowed approach (*F*_3,449_ = 6.58, *p* < 0.001), indicating that increased number of stimulation in both, VERUM and SHAM, is related to increased HR. Thus, a higher number of slow waves is likely related to an accelerated HR.

Next, we investigated whether the induced change in lowSWA (as measured by the percentage difference in lowSWA compared with the SHAM condition) correlates with the mean HR, HRV or BPW during NREM sleep, all relative to SHAM. We employed repeated measures correlations and found a significant positive correlation between the increase in lowSWA and HR (*p* < 0.001; Figure 6c), again pointing towards increased HR when more slow waves are present. However, we found no significant correlation between relative HRV (*p* = 0.17) and relative BPW (*p* = 0.27), and relative lowSWA.

## 4 DISCUSSION

In this work, we investigated the effects of auditory deep sleep stimulation on cardiovascular secondary outcomes in healthy older participants. During 2‐week VERUM (stimulation) and SHAM (no stimulation) periods, we measured cardiac autonomic parameters during sleep, such as HR and HRV to indirectly derive parasympathetic activity, and BPW, a marker estimating sympathetic activity. The primary outcome measure of lowSWA change as a response to auditory stimulation has been reported previously (Lustenberger et al., 2022). In this previous analysis, both continuous and windowed stimulation enhanced SWA. Moreover, trait like baseline SWA as measured during the first 10 min of consolidated NREM sleep predicted how individuals responded to the stimulation, with lower baseline values predicting lower slow‐wave enhancements (Lustenberger et al., 2022). Here, we show that sleep stages influence cardiac autonomic activity profiles. Because the underlying cardiovascular state changes during the night (Brandenberger et al., 2001; Jurysta et al., 2003; Viola et al., 2002), we split the night into two equal parts, and showed that averaging cardiac autonomic parameters across a complete night cancels out differences between sleep stages. Moreover, our findings illustrate that during NREM sleep, HR is elevated on nights characterized by a more pronounced lowSWA. Participants with generally higher baseline lowSWA showed increased HR, increased HRV and increased BPW across all sleep stages compared with participants with lower baseline lowSWA. We did not observe any difference in auditory stimulation on any of the cardiac autonomic parameters, except for a slight decrease in HRV for the continuous stimulation approach. However, we found the more stimulations were applied (in SHAM: only marked triggers, indicating a high slow‐wave level), the higher the HR within NREM sleep. Furthermore, there was a significant positive within‐subject correlation between SWA and HR, altogether pointing towards increased HR when slow waves are more pronounced.

### 4.1 Averaging cardiac autonomic parameters across a complete night diminishes underlying dynamics

When examining the variations in cardiac autonomic parameters within different sleep stages within a night, we found that NREM2, as opposed to NREM3, demonstrated the most pronounced cardiac autonomic recovery profile indicated through parasympathetic predominance (increase in HRV), relative sympathetic withdrawal (decrease in HRV) and decrease in HR as an outcome of the cardiac autonomic modulation. This is evident from the lowest HR, highest HRV and lowest BPW in our data. Deep NREM sleep and SWA have been repeatedly related to parasympathetic predominance (Brandenberger et al., 2001; Grimaldi et al., 2019c; Jurysta et al., 2003; Somers et al., 1993) and, thus, we expected HR to be lowest, HRV to be highest and BPW to be lowest during NREM3 sleep. However, already Tank et al. (2003) did not observe lower HR within deep sleep compared with a resting wake period. This might be explained because HR is generally decreasing with duration of sleep (Cajochen et al., 1994), and within the early morning hours there is limited NREM3 sleep alongside an increasing proportion of REM sleep (Carskadon & Dement, 2011). Furthermore, Boudreau and colleagues (Boudreau et al., 2013) showed that absolute indices for parasympathetic activity (such as HRV) are lower during NREM3 compared with, for example, NREM1; however, when considering relative indices reflecting sympathovagal balance, NREM3 sleep showed the highest parasympathetic predominance. Thus, averaging cardiac autonomic parameters across a complete night while separating NREM2 and NREM3 might reflect an inaccurate picture of the cardiac autonomic dynamics. These patterns could be emphasized when considering that NREM2 constitutes over 60% of a night's sleep, while NREM3 comprises less than 10% in adults aged 60 years and above (Ohayon et al., 2004). Another reason why our cardiac autonomic profile contradicts previous findings (Brandenberger et al., 2001; Busek et al., 2005; Grimaldi et al., 2019c; Jurysta et al., 2003; Somers et al., 1993; Tobaldini et al., 2013; Trinder et al., 2012) could be age. Cardiac autonomic activity during sleep differs between younger and older individuals, whereas especially parasympathetic activity is decreased with age (Brandenberger et al., 2003; Chen et al., 2021; Jurysta et al., 2005). This decrease is particularly occurring during NREM sleep (Chen et al., 2021) and is related to the decreased level of slow waves (Jurysta et al., 2005). However, as we only measured an older population, we cannot conclude to what extent the changed cardiac autonomic profile is because of age or because of averaging across a night. Nevertheless, the observed HRV was highest during wake periods. This may be explained because HRV (RMSSD here) was calculated based on a 5‐min moving average, and wake periods are accompanied by a large increase in HR (Sforza et al., 2000). Thus, HRV is increased through the rapid change in HR, which can be also illustratively seen in Figure 2(b). During the second part of the night, we found a general decrease in HR, an increase in HRV for REM sleep, and a decrease in BPW for NREM and REM, compared with the first part, altogether indicating stronger parasympathetic predominance during later parts of the night.

### 4.2 Baseline lowSWA level predicts cardiac autonomic regulation during sleep

Based on our previous observation that baseline SWA level, which likely is a personal trait, influences how strongly participants reacted to auditory stimulation (Lustenberger et al., 2022), we wanted to investigate whether cardiac autonomic parameters also reflect a trait between these two groups. Contrary to our expectation, weak responders generally exhibited very little NREM3 sleep and low baseline SWA (Lustenberger et al., 2022), and showed decreased HR, decreased HRV and decreased BPW compared with strong responders, indicating rather increased parasympathetic predominance. Comparing the strong responder and weak responder groups, there are more males in the weak responder group with a higher average age. Generally, aging leads to a decrease in deep NREM sleep and SWA (Carskadon & Dement, 2011; Landolt et al., 1996), but females seem to preserve higher SWA levels up until older age (e.g. summarized in Carrier et al., 2017). Both may explain the higher proportion of females and younger age of the strong responder group. Furthermore, HR within sleep in middle‐aged males was reported to be elevated compared with a younger cohort (Jurysta et al., 2005), whereas aging is related to HRV in a U‐shaped fashion (Almeida‐santos et al., 2016). Additionally, sex seems to influence HRV, some propose females to have increased HRV up until old age (Almeida‐santos et al., 2016) whereas others report sex differences in HRV to be diminished with aging (Bonnemeier et al., 2003). There seems to be a tendency for increased sympathetic and decreased parasympathetic activity with aging (Almeida‐santos et al., 2016), possibly because of the age‐related decline in SWA (Brandenberger et al., 2003).

Hence, the increased HR and decreased BPW indicating decreased sympathetic activity cannot be solely explained by sex and age differences between the two groups. However, the found interaction between sleep stage and the markers for parasympathetic and sympathetic activity suggests that baseline lowSWA level (respond index) differently influences the dynamics of the cardiac autonomic modulation during sleep. Therefore, baseline slow‐wave level seems to significantly predict cardiac autonomic parameters.

### 4.3 Auditory stimulation slightly decreases HRV for the continuous approach only but does not influence other cardiac autonomic parameters

We next explored whether auditory stimulation, which successfully enhanced slow waves in particularly the group of strong responders (Lustenberger et al., 2022), also affects cardiac autonomic modulation. Surprisingly, we found no effect of slow‐wave enhancement either within the windowed or the continuous approach on HR and BPW. Contrary to the previously reported increase in HRV while auditory stimulation was applied (Diep et al., 2022; Grimaldi et al., 2019c; Huwiler et al., 2022), HRV was even significantly decreased for the continuous approach. Our participant group was older compared with previous groups, which might be one possible explanation. However, initial work on continuous auditory stimulation proposed that some mechanisms counteract the development of hypersynchrony within the brain as long as slow waves were present (Ngo et al., 2015), thereby limiting the effects of evoking slow waves. To that end, the continuous stimulation might also interfere with dynamic cardiac processes observed while administering auditory stimulation. Namely, the observed biphasic HR response in a windowed stimulation design (Huwiler et al., 2022) likely induced by tone‐evoked K‐complexes (de Zambotti et al., 2016) might be interrupted because of a refractory period of K‐complexes (Bernardi et al., 2018). Thereby, HRV could decrease. Nevertheless, the decrease in HRV is small and whether this would translate into a functionally relevant post‐sleep effect needs further investigation.

The absence of an effect of auditory stimulation on any other cardiac autonomic parameter could potentially be caused by again averaging these parameters for each epoch and across times when stimulations were applied. A previous study applying auditory stimulation also observed no difference between stimulation and SHAM in HR or RMSSD as an HRV marker averaged across NREM sleep cycles (Grimaldi et al., 2019). Thus, the direct effects of the auditory slow‐wave enhancement may be dynamic (see our previous work describing HR acceleration followed by HR deceleration during times of stimulation; Huwiler et al., 2022). Similarly, BPW might also be affected dynamically, which cannot be observed by averaging across epochs. Altogether, the 20‐s epoch resolution for HR, or the even longer period for HRV and BPW, for our cardiac autonomic parameters is likely not sufficient to observe how auditory slow‐wave enhancement changes cardiac autonomic regulation directly.

### 4.4 HR increases when slow waves are more pronounced during NREM sleep

Most research projects focused on HRV parameters as markers for sympathovagal balance, sympathetic activity and parasympathetic activity, and neglected to establish the overall output of autonomic functioning (Stein & Pu, 2012) in relation to EEG changes. Besides the respond index, which reflects baseline SWA influencing cardiac autonomic modulation and therefore suggesting higher HR to be a trait, we found intra‐ and inter‐night indications pointing towards the fact that within NREM sleep, higher slow‐wave levels are related to increased HR. However, we found no correlation between either HRV or BPW and lowSWA within NREM sleep. Intra‐night, the number of stimulations significantly influenced HR within the VERUM and notably within the SHAM condition, suggesting that a higher number of detected slow waves, which are needed for stimulations to occur, increase HR. SWA is highest during the first sleep cycles and, during these times, also HR is higher compared with the later parts of the night (Cajochen et al., 1994). These observations would point towards the positive relationship between HR and lowSWA to be a byproduct of the homeostatic SWA regulation within sleep. However, the positive correlation of HR and lowSWA between different nights within the same participants (inter‐night) supports the evidence that increases in slow waves are coupled with increases in HR. Nevertheless, the increased HR while SWA is highest does not fit with the previously reported positive correlation between SWA and autonomic parameters (Brandenberger et al., 2001; Somers et al., 1993), indicating increased parasympathetic predominance when high SWA levels are present.

HR reflects the output of the summation of cardiac autonomic activity during sleep. However, some of these autonomic processes could also reflect a general trait of each person. For instance, the intra‐night variability in lowSWA and cardiac autonomic parameters might be related to homeostatic processes not only increasing lowSWA but also influencing cardiac autonomic function. Moreover, circadian processes have been shown to influence general HR levels during sleep (Vandewalle et al., 2007), such as HR during an afternoon nap is increased compared with night‐time sleep (Whitehurst et al., 2018). Additionally, the parasympathetic nervous system has been reported to be influenced by circadian rhythms, whereas the sympathetic nervous system is rather influenced by sleep itself (Burgess et al., 1997). Both could partially explain why we found a positive correlation between lowSWA with HR within NREM sleep. Besides circadian influences, sleep continuity is another factor influencing HR dynamics reflected by higher HR after brief arousals or wake ups (Burgess et al., 1997), most likely caused by increased sympathetic activity (Sforza et al., 2000). Altogether, these factors might even have stronger effects on HR than underlying sleep stage dynamics, such as different levels of lowSWA during sleep, but SWA is likely superimposing circadian variation of the cardiac autonomic parameters.

We nevertheless found several implications that the observed increase in HR when slow waves were prevailing cannot only be explained by circadian or homeostatic processes. Particularly the extent of lowSWA enhancement by auditory stimulation was related to higher average HR within NREM sleep compared with the SHAM condition. Recent evidence in mice points towards induced increases in SWA leading to increased HR (Forero et al., 2023), corresponding to our findings in humans. Furthermore, night‐time autonomic activity was suggested to drive overnight memory consolidation (Whitehurst et al., 2016). Although lower HR is associated with more rested sympathovagal balance, brief increases in HR during NREM sleep periods, for example, related to K‐complexes, have been reported (Carro‐Domínguez et al., 2023; de Zambotti et al., 2016). These biphasic HR responses were suggested to be involved in maintaining cardiovascular homeostasis during sleep (de Zambotti et al., 2016; Huwiler et al., 2023). Along these lines, our group recently demonstrated the strength of this cardiovascular activation response predicts post‐sleep cardiac function (Alessandrelli et al., 2024), which is increased after auditory stimulated nights in healthy middle‐aged participants (Huwiler et al., 2023). Similarly, Chen and colleagues (Chen et al., 2020) proposed that the coupling between HR and frontal SWA benefits working memory performance. Specifically, they reported that short accelerations of HR were occurring concurrently with increases in SWA, and these autonomic central coupling events predicted memory consolidation (Chen et al., 2020; Naji et al., 2018). Therefore, these reported temporal bursts of HR (Chen et al., 2020; Naji et al., 2018) could be a potential explanation for the found positive correlation between SWA and HR, although this cannot be assessed because of the insufficient temporal resolution of our HR data. These slight increases in HR as observed in our study might thus not be disadvantageous to the cardiovascular system. Nevertheless, whether these effects are also beneficial in a slightly older population remains to be investigated.

### 4.5 Limitations

Because this trial was fully conducted at each participant's home, we used a CE‐certified wearable with limited access to raw beat‐to‐beat data. Thus, the cardiac autonomic parameters are calculated on a moving average basis and do not allow for dynamic analysis of the cardiovascular reaction during specific times of stimulation. Because BPW is a novel marker for sympathetic activity introduced by Biovotion and has not been extensively validated, these results might be interpreted with caution. Furthermore, the sex and age distribution of strong responders and weak responders are not equal, particularly intensified through excluding one female participant in the weak responder groups. This might be the case because of sex and age differences influencing the baseline SWA, which is in turn predicting whether participants are rather strong responders or weak responders. Larger‐scale studies should further explore the importance of age and gender to verify our observed findings as our number of participants is not large enough to conduct sex‐ or age‐specific analysis.

## 5 CONCLUSION

We confirm that cardiac autonomic parameters differ between sleep stages; however, accounting for the time of the night is crucial. Averaging these parameters across a complete night can mask differences between sleep stages, because of underlying physiological changes across the night. We further demonstrate that baseline lowSWA indicates distinctive cardiac autonomic profiles. Participants with higher baseline lowSWA show higher HR, higher HRV and higher BPW levels during sleep, pointing towards both increased parasympathetic and sympathetic activity compared with participants with lower baseline lowSWA. We furthermore showed an inter‐ and intra‐night within‐subject positive relationship between lowSWA and HR, which aligns with a more pronounced increase in lowSWA to auditory stimulation predicting increases in HR. Altogether, our results indicate increased HR when lowSWA is prevailing. Nevertheless, subsequent studies should also investigate the long‐term post‐sleep effects on cardiac autonomic parameters.

## AUTHOR CONTRIBUTIONS

**Stephanie Huwiler:** Investigation; validation; formal analysis; visualization; writing – review and editing; writing – original draft; software; data curation; conceptualization. **M. Laura Ferster:** Writing – review and editing; methodology; formal analysis. **Luzius Brogli:** Formal analysis; writing – review and editing; software. **Reto Huber:** Data curation; conceptualization; writing – review and editing. **Walter Karlen:** Funding acquisition; supervision; conceptualization; methodology; writing – review and editing. **Caroline Lustenberger:** Formal analysis; conceptualization; resources; project administration; funding acquisition; writing – original draft; writing – review and editing; supervision; data curation.

## FUNDING INFORMATION

This study was funded in part by the Schweizerische Hirn Stiftung, Muriel Nikles and the ETH Zurich Foundation, and the Swiss National Science Foundation (P3P3PA_171525 and PZ00P3_179795 to C.L.).

## CONFLICT OF INTEREST STATEMENT

R.H. and W.K. are founders and shareholders of Tosoo AG that has licensed the technology used in this work. C.L. is a member of the Scientific Advisory Board of Emma Sleep GmbH, which is not related to this work.

## PATIENT CONSENT STATEMENT

All participants provided written informed consent before participation and received monetary compensation for their participation.

## Supporting information


**DATA S1.** Supporting information.

## Data Availability

The data that support the findings of this study are available on request from the corresponding author. The data are not publicly available due to privacy or ethical restrictions.

## References

[jsr14328-bib-0001] Alessandrelli, G. , Huwiler, S. , Bernardi, G. , Carro‐domínguez, M. , & Stich, F. (2024). Cardiovascular responses to natural and auditory evoked slow waves predict post‐sleep cardiac function. biorxiv, 1–20. 10.1101/2024.05.03.592377 PMC13619023 42220219

[jsr14328-bib-0002] Almeida‐santos, M. A. , Barreto‐filho, J. A. , Oliveira, J. L. M. , Reis, F. P. , da Cunha Oliveira, C. C. , & Sousa, A. C. S. (2016). Aging, heart rate variability and patterns of autonomic regulation of the heart. Archives of Gerontology and Geriatrics, 63, 1–8. 10.1016/j.archger.2015.11.011 26791165

[jsr14328-bib-0003] Bernardi, G. , Siclari, F. , Handjaras, G. , Riedner, B. A. , & Tononi, G. (2018). Local and widespread slow waves in stable NREM sleep: Evidence for distinct regulation mechanisms. Frontiers in Human Neuroscience, 12, 1–13. 10.3389/fnhum.2018.00248 29970995 PMC6018150

[jsr14328-bib-0004] Bonnemeier, H. , Uwekh, W. , Brandes, A. , Kluge, N. , Katus, H. A. , & Richardt, G. (2003). Circadian profile of cardiac autonomic nervous modulation in healthy subjects: Differing effects of aging and gender on heart rate variability. Journal of Cardiovascular Electrophysiology, 14(8), 791–799. 12890036 10.1046/j.1540-8167.2003.03078.x

[jsr14328-bib-0005] Boudreau, P. , Yeh, W. H. , Dumont, G. A. , & Boivin, D. B. (2013). Circadian variation of heart rate variability across sleep stages. Sleep, 36(12), 1919–1928. 10.5665/sleep.3230 24293767 PMC3825442

[jsr14328-bib-0006] Brandenberger, G. , Ehrhart, J. , Piquard, F. , & Simon, C. (2001). Inverse coupling between ultradian oscillations in delta wave activity and heart rate variability during sleep. Clinical Neurophysiology, 112(6), 992–996. 10.1016/S1388-2457(01)00507-7 11377256

[jsr14328-bib-0007] Brandenberger, G. , Viola, A. U. , Ehrhart, J. , Charloux, A. , Geny, B. , Piquard, F. , & Simon, C. (2003). Age‐related changes in cardiac autonomic control during sleep. Journal of Sleep Research, 12(3), 173–180. 10.1046/j.1365-2869.2003.00353.x 12941056

[jsr14328-bib-0008] Brunner, D. P. , Vasko, R. C. , Detka, C. S. , Monahan, J. P. , Reynolds, C. F. , & Kupfer, D. J. (1996). Muscle artifacts in the sleep EEG: Automated detection and effect on all‐night EEC power spectra. Journal of Sleep Research, 5(3), 155–164. 10.1046/j.1365-2869.1996.00009.x 8956205

[jsr14328-bib-0009] Burgess, H. J. , Trinder, J. , Kim, Y. , & Luke, D. (1997). Sleep and circadian influences on cardiac autonomic nervous system activity. American Journal of Physiology‐Heart and Circulatory Physiology, 273(4 42‐4), 1761–1768. 10.1152/ajpheart.1997.273.4.h1761 9362241

[jsr14328-bib-0010] Busek, P. , Vanková, J. , Opavský, J. , et al. (2005). Spectral analysis of the heart rate variability in sleep. Physiological Research, 54(4), 369–376. 10.1515/ijdhd-2014-0025 15588154

[jsr14328-bib-0011] Cabiddu, R. , Cerutti, S. , Viardot, G. , Werner, S. , & Bianchi, A. M. (2012). Modulation of the sympatho‐vagal balance during sleep: Frequency domain study of heart rate variability and respiration. Frontiers in Physiology, 3, 1–10. 10.3389/fphys.2012.00045 22416233 PMC3299415

[jsr14328-bib-0012] Cajochen, C. , Pischke, J. , Aeschbach, D. , & Borbély, A. A. (1994). Heart rate dynamics during human sleep. Physiology & Behavior, 55(4), 769–774. 10.1016/0031-9384(94)90058-2 8190808

[jsr14328-bib-0013] Carrier, J. , Semba, K. , Deurveilher, S. , Drogos, L. , Cyr‐Cronier, J. , Lord, C. , & Sekerovick, Z. (2017). Sex differences in age‐related changes in the sleep‐wake cycle. Frontiers in Neuroendocrinology, 47, 66–85. 10.1016/j.yfrne.2017.07.004 28757114

[jsr14328-bib-0014] Carro‐Domínguez, M. , Huwiler, S. , Oberlin, S. , Oesch, T. L. , Badii, G. , Lüthi, A. , Wenderoth, N. , Meissner, S. N. , & Lustenberger, C. (2023). Pupil size reveals arousal level dynamics in human sleep. bioRxiv, 2023‐07. 10.1101/2023.07.19.549720 PMC11871316 40021662

[jsr14328-bib-0015] Carskadon, M. A. , & Dement, W. C. (2011). Normal human sleep: An Overview. In M. H. Kryger , T. Roth , & W. C. Dement (Eds.), Principles and Practice of Sleep Medicine (5th ed., pp. 16–26). Elsevier Saunders.

[jsr14328-bib-0016] Chen, P. C. , Sattari, N. , Whitehurst, L. N. , & Mednick, S. C. (2021). Age‐related losses in cardiac autonomic activity during a daytime nap. Psychophysiology, 58(7), 1–17. 10.1111/psyp.13701 PMC8041919 33048396

[jsr14328-bib-0018] Chen, P. C. , Whitehurst, L. N. , Naji, M. , & Mednick, S. C. (2020). Autonomic/central coupling benefits working memory in healthy young adults. Neurobiology of Learning and Memory, 173, 107267. 10.1016/j.nlm.2020.107267 32535198

[jsr14328-bib-0019] Colten, H. R. , & Altevogt, B. M. (2006). Sleep Disorders and Sleep Deprivation: An Unmet Public Health Problem. National Academies Press. 10.17226/11617 20669438

[jsr14328-bib-0020] de Zambotti, M. , Willoughby, A. R. , Franzen, P. L. , Clark, D. B. , Baker, F. C. , & Colrain, I. M. (2016). K‐complexes: Interaction between the central and autonomic nervous systems during sleep. Sleep, 39(5), 1129–1137. 10.5665/sleep.5770 26856907 PMC4835312

[jsr14328-bib-0021] Dick, R. , Penzel, T. , Fietze, I. , Partinen, M. , Hein, H. , & Schulz, J. (2010). AASM standards of practice compliant validation of actigraphic sleep analysis from somnowatch™ versus polysomnographic sleep diagnostics shows high conformity also among subjects with sleep disordered breathing. Physiological Measurement, 31(12), 1623–1633. 10.1088/0967-3334/31/12/005 21071830

[jsr14328-bib-0022] Diep, C. , Ftouni, S. , Drummond, S. P. A. , Garcia‐Molina, G. , & Anderson, C. (2022). Heart rate variability increases following automated acoustic slow wave sleep enhancement. Journal of Sleep Research, 31(5), e13545. 10.1111/jsr.13545 35080060

[jsr14328-bib-0023] Diep, C. , Ftouni, S. , Manousakis, J. E. , Nicholas, C. L. , Drummond, S. P. A. A. , & Anderson, C. (2020). Acoustic slow wave sleep enhancement via a novel, automated device improves executive function in middle‐aged men. Sleep, 43(1), 1–11. 10.1093/sleep/zsz197 31691831

[jsr14328-bib-0024] Espiritu, J. R. D. (2008). Aging‐related sleep changes. Clinics in Geriatric Medicine, 24(1), 1–14. 18035227 10.1016/j.cger.2007.08.007

[jsr14328-bib-0025] Fernández‐Varela, I. , Alvarez‐Estevez, D. , Hernández‐Pereira, E. , & Moret‐Bonillo, V. (2017). A simple and robust method for the automatic scoring of EEG arousals in polysomnographic recordings. Computers in Biology and Medicine, 87, 77–86. 28554078 10.1016/j.compbiomed.2017.05.011

[jsr14328-bib-0026] Ferster, M. L. , Lustenberger, C. , & Karlen, W. (2019). Configurable mobile system for autonomous high‐quality sleep monitoring and closed‐loop acoustic stimulation. IEEE Sensors Letters, 3(5), 6000904. 10.1109/LSENS.2019.2914425

[jsr14328-bib-0027] Forero, A. O. , Foustoukos, G. , Cardis, R. , Cherrad, N. , Devenoges, C. , Fernandez, L. M. , & Luthi, A. (2023). Locus coeruleus activity fluctuations set a non‐reducible timeframe for mammalian NREM‐REM sleep cycles. bioRxiv, 184759, 1–40.

[jsr14328-bib-0028] Grandner, M. A. , Alfonso‐Miller, P. , Fernandez‐Mendoza, J. , Shetty, S. , Shenoy, S. , & Combs, D. (2016). Sleep: Important considerations for the prevention of cardiovascular disease. Current Opinion in Cardiology, 31(5), 551–565. 10.1097/HCO.0000000000000324 27467177 PMC5056590

[jsr14328-bib-0029] Grimaldi, D. , Papalambros, N. A. , Reid, K. J. , Abbott, S. M. , Malkani, R. G. , Gendy, M. , Iwanaszko, M. , Braun, R. I. , Sanchez, D. J. , Paller, K. A. , & Zee, P. C. (2019). Strengthening sleep–autonomic interaction via acoustic enhancement of slow oscillations. Sleep, 42(5), zsz036. 10.1093/sleep/zsz036 30753650 PMC7729207

[jsr14328-bib-0030] Huwiler, S. , Carro Dominguez, M. , Huwyler, S. , Kiener, L. , Stich, F. M. , Sala, R. , Aziri, F. , Trippel, A. , Schmied, C. , Huber, R. , Wenderoth, N. , & Lustenberger, C. (2022). Effects of auditory sleep modulation approaches on brain oscillatory and cardiovascular dynamics. Sleep, 45(9), 1–36. 10.1093/sleep/zsac155 PMC9453626 35793672

[jsr14328-bib-0031] Huwiler, S. , Carro‐Domínguez, M. , Stich, F. M. , Sala, R. , Aziri, F. , Trippel, A. , Ryf, T. , Markendorf, S. , Niederseer, D. , Bohm, P. , Stoll, G. , Laubscher, L. , Thevan, J. , Spengler, C. M. , Gawinecka, J. , Osto, E. , Huber, R. , Wenderoth, N. , Schmied, C. , & Lustenberger, C. (2023). Auditory stimulation of sleep slow waves enhances left ventricular function in humans. European Heart Journal, 44(40), 4288–4291. 10.1093/eurheartj/ehad630 37794725 PMC10590124

[jsr14328-bib-0032] Jurysta, F. , Van De Borne, P. , Lanquart, J. P. , Migeotte, P. F. , Degaute, J. P. , Dumont, M. , & Linkowski, P. (2005). Progressive aging does not alter the interaction between autonomic cardiac activity and delta EEG power. Clinical Neurophysiology, 116(4), 871–877. 10.1016/j.clinph.2004.10.005 15792896

[jsr14328-bib-0033] Jurysta, F. , Van De Borne, P. , Migeotte, P. F. , Dumont, M. , Lanquart, J. P. , Degaute, J. P. , & Linkowski, P. (2003). A study of the dynamic interactions between sleep EEG and heart rate variability in healthy young men. Clinical Neurophysiology, 114(11), 2146–2155. 10.1016/S1388-2457(03)00215-3 14580613

[jsr14328-bib-0034] Landolt, H. P. , Dijk, D. J. , Achermann, P. , & Borbély, A. A. (1996). Effect of age on the sleep EEG: Slow‐wave activity and spindle frequency activity in young and middle‐aged men. Brain Research, 738(2), 205–212. 10.1016/S0006-8993(96)00770-6 8955514

[jsr14328-bib-0035] Lustenberger, C. , Ferster, M. L. , Huwiler, S. , Brogli, L. , Werth, E. , Huber, R. , & Karlen, W. (2022). Auditory deep sleep stimulation in older adults at home: A randomized crossover trial. Communication & Medicine, 2(1), 30. 10.1038/s43856-022-00096-6 PMC9053232 35603302

[jsr14328-bib-0036] Lustenberger, C. , Wehrle, F. , Tüshaus, L. , Achermann, P. , & Huber, R. (2015). The multidimensional aspects of sleep spindles and their relationship to word‐pair memory consolidation. Sleep, 38(7), 1093–1103. 10.5665/sleep.4820 25845686 PMC4481015

[jsr14328-bib-0037] Mancia, G. (1993). Autonomic modulation of the cardiovascular system during sleep. The New England Journal of Medicine, 328(5), 347–349. 10.1056/nejm199302043280511 8419822

[jsr14328-bib-0038] Mander, B. A. , Rao, V. , Lu, B. , Saletin, J. M. , Lindquist, J. R. , Ancoli‐Israel, S. , Jagust, W. , & Walker, M. P. (2013). Prefrontal atrophy, disrupted NREM slow waves and impaired hippocampal‐dependent memory in aging. Nature Neuroscience, 16(3), 357–364. 10.1038/nn.3324 23354332 PMC4286370

[jsr14328-bib-0039] Medic, G. , Wille, M. , & Hemels, M. E. H. (2017). Short‐ and long‐term health consequences of sleep disruption. Nature and Science of Sleep, 9, 151–161. 10.2147/NSS.S134864 PMC5449130 28579842

[jsr14328-bib-0040] Naji, M. , Krishnan, G. P. , McDevitt, E. A. , Bazhenov, M. , & Mednick, S. C. (2018). Coupling of autonomic and central events during sleep benefits declarative memory consolidation. Neurobiology of Learning and Memory, 2019(157), 139–150. 10.1016/j.nlm.2018.12.008 PMC6425961 30562589

[jsr14328-bib-0041] Ngo, H. V. V. , Martinetz, T. , Born, J. , Mölle, M. , & Molle, M. (2013). Auditory closed‐loop stimulation of the sleep slow oscillation enhances memory. Neuron, 78(3), 545–553. 10.1016/j.neuron.2013.03.006 23583623

[jsr14328-bib-0042] Ngo, H. V. V. , Miedema, A. , Faude, I. , Martinetz, T. , Mölle, M. , & Born, J. (2015). Driving sleep slow oscillations by auditory closed‐loop stimulation—A self‐limiting process. The Journal of Neuroscience, 35(17), 6630–6638. 10.1523/JNEUROSCI.3133-14.2015 25926443 PMC4412888

[jsr14328-bib-0043] North, B. J. , & Sinclair, D. A. (2012). The intersection between aging and cardiovascular disease. Circulation Research, 110(8), 1097–1108. 10.1161/CIRCRESAHA.111.246876 22499900 PMC3366686

[jsr14328-bib-0044] Ohayon, M. M. , Carskadon, M. A. , Guilleminault, C. , & Vitiello, M. V. (2004). Meta‐analysis of quantitative sleep parameters from childhood to old age in healthy individuals: Developing normative sleep values across the human lifespan. Sleep, 27(7), 1255–1273. 10.1093/sleep/27.7.1255 15586779

[jsr14328-bib-0045] Sforza, E. , Jouny, C. , & Ibanez, V. (2000). Cardiac activation during arousal in humans: Further evidence for hierarchy in the arousal response. Clinical Neurophysiology, 111(9), 1611–1619. 10.1016/S1388-2457(00)00363-1 10964073

[jsr14328-bib-0046] Somers, V. K. , Dyken, M. E. , Mark, A. L. , & Abboud, F. M. (1993). Sympathetic‐nerve activity during sleep in Normal subjects. The New England Journal of Medicine, 328(5), 303–307. 10.1056/NEJM199302043280502 8419815

[jsr14328-bib-0047] Stein, P. K. , & Pu, Y. (2012). Heart rate variability, sleep and sleep disorders. Sleep Medicine Reviews, 16(1), 47–66. 10.1016/j.smrv.2011.02.005 21658979

[jsr14328-bib-0048] Tank, J. , Diedrich, A. , Hale, N. , Niaz, F. E. , Furlan, R. , Robertson, R. M. , & Mosqueda‐Garcia, R. (2003). Relationship between blood pressure, sleep K‐complexes, and muscle sympathetic nerve activity in humans. American Journal of Physiology‐Regulatory, Integrative and Comparative Physiology, 285(1), R208–R214. 10.1152/ajpregu.00013.2003 12793998

[jsr14328-bib-0049] Tobaldini, E. , Nobili, L. , Strada, S. , Casali, K. R. , Braghiroli, A. , & Montano, N. (2013). Heart rate variability in normal and pathological sleep. Frontiers in Physiology, 4, 1–11. 10.3389/fphys.2013.00294 24137133 PMC3797399

[jsr14328-bib-0050] Trinder, J. , Waloszek, J. , Woods, M. J. , & Jordan, A. S. (2012). Sleep and cardiovascular regulation. Pflügers Archiv‐European Journal of Physiology, 463(1), 161–168. 10.1007/s00424-011-1041-3 22038322

[jsr14328-bib-0051] Vandewalle, G. , Middleton, B. , Rajaratnam, S. M. W. , Stone, B. M. , Thorleifsdottir, B. , Arendt, J. , & Dijk, D. J. (2007). Robust circadian rhythm in heart rate and its variability: Influence of exogenous melatonin and photoperiod. Journal of Sleep Research, 16(2), 148–155. 10.1111/j.1365-2869.2007.00581.x 17542944

[jsr14328-bib-0052] Viola, A. U. , Simon, C. , Ehrhart, J. , Geny, B. , Piquard, F. , Muzet, A. , & Brandenberger, G. (2002). Sleep processes exert a predominant influence on the 24‐h profile of heart rate variability. Journal of Biological Rhythms, 17(6), 539–547. 10.1177/0748730402238236 12465887

[jsr14328-bib-0053] Whitehurst, L. N. , Cellini, N. , McDevitt, E. A. , Duggan, K. A. , & Mednick, S. C. (2016). Autonomic activity during sleep predicts memory consolidation in humans. Proceedings of the National Academy of Sciences, 113(26), 7272–7277. 10.1073/pnas.1518202113 PMC4932927 27298366

[jsr14328-bib-0054] Whitehurst, L. N. , Naji, M. , & Mednick, S. C. (2018). Comparing the cardiac autonomic activity profile of daytime naps and nighttime sleep. Neurobiology of Sleep and Circadian Rhythms, 5, 52–57. 10.1016/j.nbscr.2018.03.001 31236511 PMC6584676

