# Supplementary material for: Sleep and cardiac autonomic modulation in older adults: Insights from an at‐home study with auditory deep sleep stimulation
Source: J Sleep Res. 2024 Sep 2;34(2):e14328. doi: 10.1111/jsr.14328 (PMC11911050; doi:10.1111/jsr.14328)
Supplement: Supplementary file 1 — DATA S1. Supporting information. [file JSR-34-e14328-s001.docx]

**Appendix - Sleep and cardiac autonomic modulation in older adults: insights from an at-home study with auditory deep sleep stimulation**

**
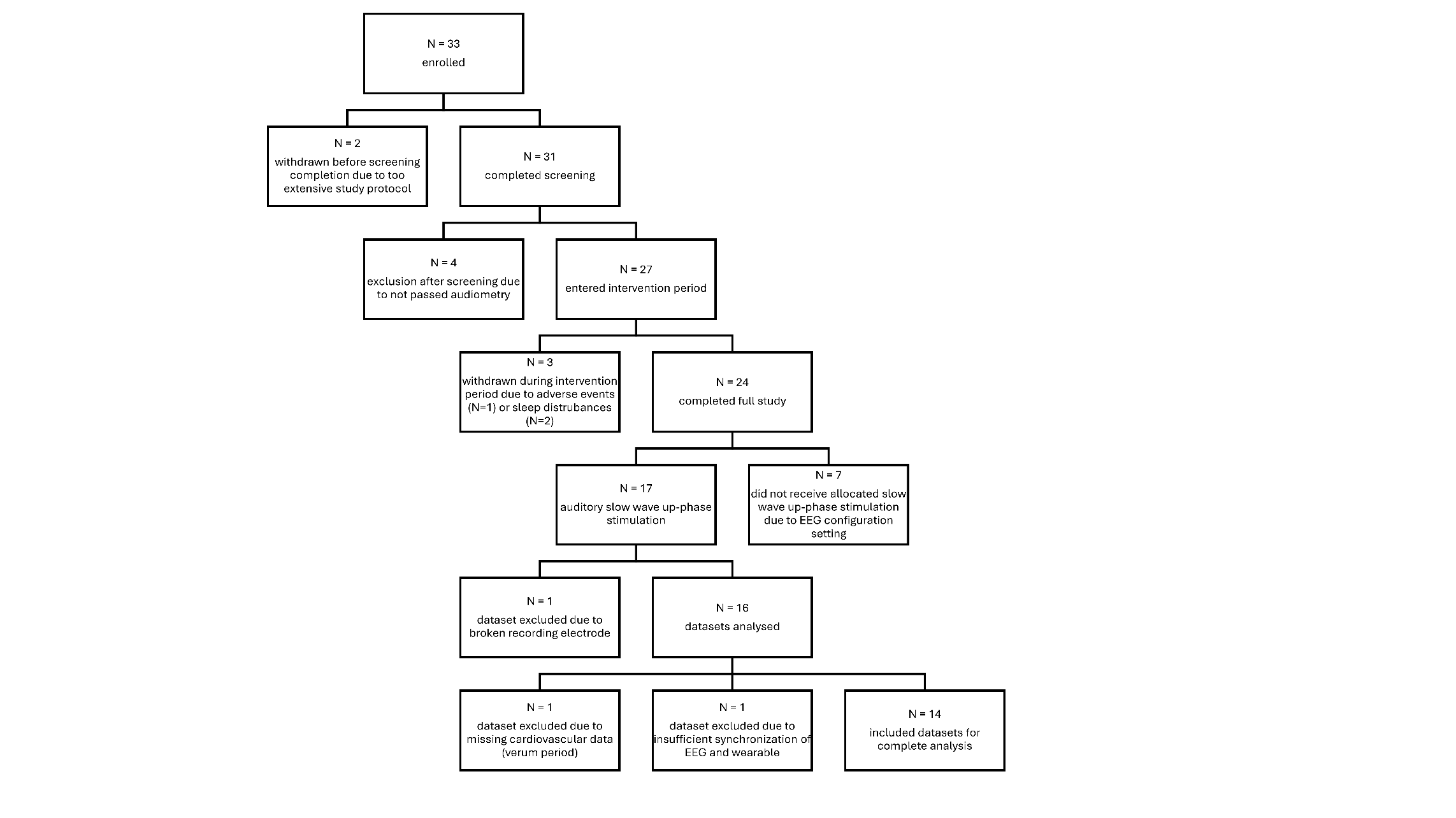
**

**Supplementary Figure 1: CONSORT flow diagram of included participants of the randomized-controlled trial and how datasets for this work were selected.**


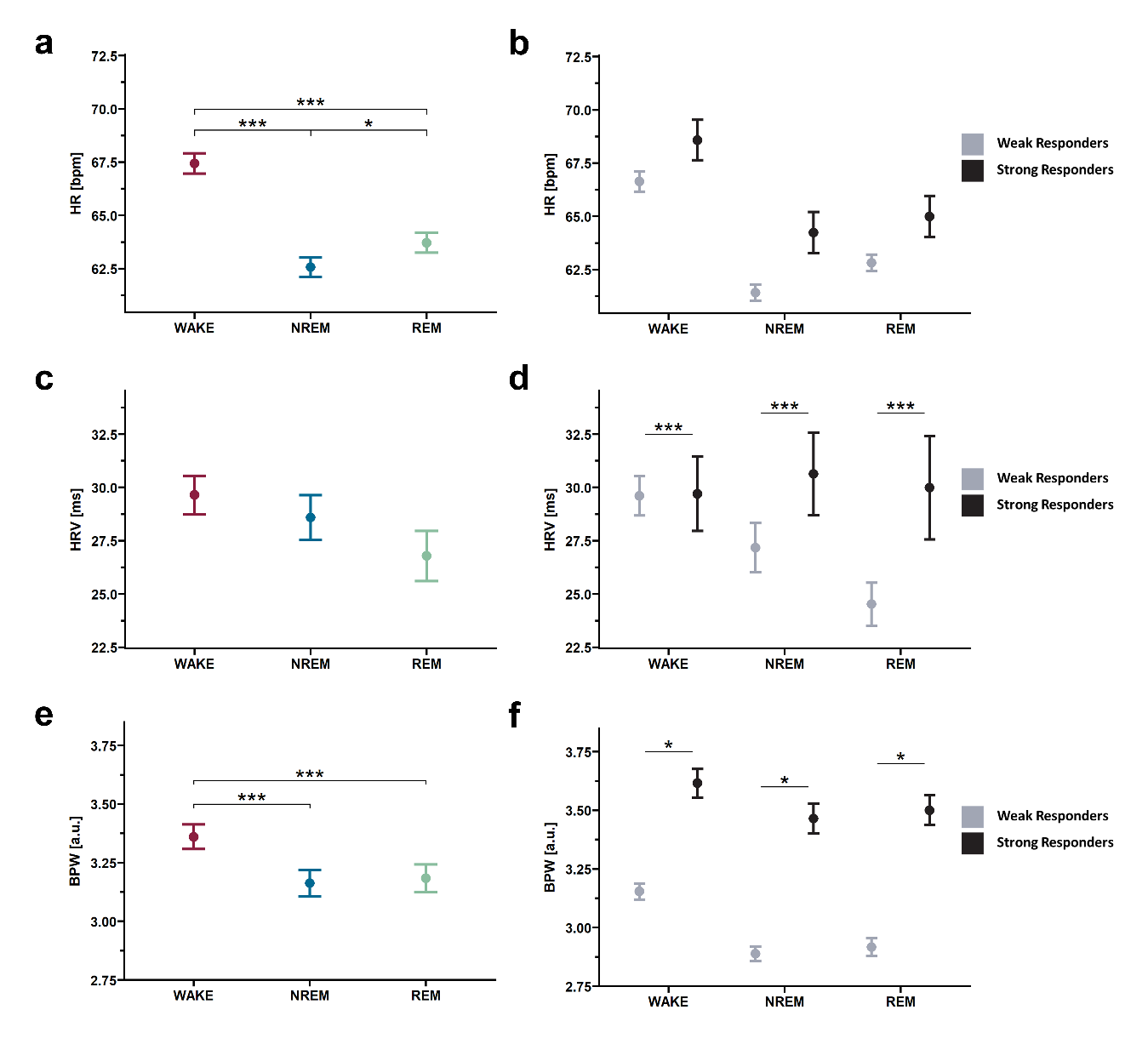


**Supplementary Figure 2:** **Sleep stage differences for heart rate (HR), HR variability (HRV) and blood pulse wave (BPW). a,c,e:** Post-hoc pairwise comparisons between sleep stage for the variables HR, HRV (calculated as RMSSD), and BPW) across the complete night were derived from linear mixed-effect models entering sleep stage and night as fixed factors, and the nested random factors subject and condition. Included sleep stages are wake, non-rapid eye movement (NREM) taken NREM 2 and NREM3 together, and rapid-eye movement sleep. **B,d,f:** HR, HRV, and BPW within each sleep stage, separated for responders and non-responders. Shown p-values show the interaction between sleep stage and respond index of the linear-mixed effect models. All data is presented as mean ± standard error of the mean. ***: p < 0.001, **: p < 0.01, *: p < 0.05.

**Supplementary Table 1: Overview of included subjects and nights for analysis**

|  | **Number Subjects** | **Number Nights** |
| --- | --- | --- |
| SHAM ON | 14 | 72 |
| SHAM ONOFF | 14 | 67 |
| VERUM ON | 14 | 67 |
| VERUM ONOFF | 13 | 63 |
